# One Step Histological Detection and Staining of the PTEN Tumor Suppressor Protein by a Single Strand DNA

**DOI:** 10.3390/diagnostics11020171

**Published:** 2021-01-26

**Authors:** Gloria Longinotti, Gabriel Ybarra, Susana Vighi, Claudia Perandones, Javier Montserrat, Juan Sebastian Yakisich, Mariano Grasselli, Martin Radrizzani

**Affiliations:** 1 Nanomateriales Funcionales, INTI-Micro y Nanotecnologías, Instituto Nacional de Tecnología Industrial (INTI), Av. Gral. Paz 5445, San Martín B1650WAB, Argentina; glorial@inti.gob.ar (G.L.); gybarra@inti.gob.ar (G.Y.); 2 Centro de Anatomía Patológica, Ciudad de la Paz 353, Buenos Aires C1426AGE, Argentina; anatomia.patologica@susanavighi.com.ar; 3 Direction of the A.N.L.I.S., Directorate National Administration of Laboratories and Institutes of Health “Dr. Carlos G. Malbrán”, Av. Vélez Sarsfield 563, Buenos Aires C1282AFF, Argentina; cperandones@anlis.gob.ar; 4 Instituto de Ciencias, Universidad Nacional de General Sarmiento, J. M. Gutiérrez 1150, Los Polvorines B1613GSX, Argentina; jmontser@campus.ungs.edu.ar; 5 Department of Pharmaceutical Sciences, School of Pharmacy, Hampton University, Hampton, VA 23693, USA; juan.yakisich@hamptonu.edu; 6 Laboratorio de Materiales Biotecnológicos (LaMaBio), Departamento de Ciencia y Tecnología, Universidad Nacional de Quilmes, GBE yB, Grupo Vinculado IMBICE-CONICET, Roque Sáenz Peña 352, Buenos Aires B1876BXDl, Argentina; Mariano.grasselli@unq.edu.ar; 7 Laboratorio de Neurología y Citogenética Molecular, (CESyMA), Escuela de Ciencia y Técnica, Universidad Nacional de San Martín, Av. Gral. Paz 5445, San Martín B1650WAB, Argentina

**Keywords:** aptamer, histochemistry, PTEN, DNAzyme-peroxidase, endometrium

## Abstract

Antibodies are the most used technological tool in histochemistry. However, even with monoclonal antibodies, their standardization is difficult due to variation of biological systems as well as to variability due to the affinity and amplification of the signal arising from secondary peroxidase detection systems. In this article we combined two synthetic molecules to facilitate the standardization of a detection protocol of protein markers in histological sections. The first molecule was an aptamer, a 50-base single-stranded DNA fragment, which recognizes a PTEN tumor suppressor. The second molecule used was also another single stranded 18-base aptamer DNA fragment, which forms a quadruplex structure guanine box. This G-quadruplex recognizes and attaches a molecule of hemin, increasing the catalytic capacity for the hydrogen peroxide. Our results show how the correct structural design of DNA combining an aptamer together with the peroxidase-like DNAzyme allows to detect proteins in histological sections. This tool offers the standardization of the detection of prognostic markers in cancer, in quality and quantity, due to its synthetic nature and its 1:1 antigen:enzyme ratio. This is the first time that reproducible results have been presented in histological sections staining a cancer marker using a single-stranded DNA molecule with dual function.

## 1. Introduction

Aptamers could be any natural, synthetic, or modified polymer, most common nucleic acid and peptides that bind to a specific target molecule. Aptamers are highly resistant to degradation and can be chemically synthesized at a relatively low cost with high reproducibility. They can be customized to incorporate chemical modifications and can be joined with affinity tags or labels. [1,2,3,4,5]. Aptamers are a rising technology applied for detecting targets using random nucleotides with specific binding affinity. Target ligands for aptamers can be any molecule, including toxins and non-immunogenic agents. In August 1990, two groups proposed an inverse method for aptamer selection by suggesting that there would be an oligonucleotide that could be expected to recognize the target chosen if there is a large enough combinatorial library. One group used short sequences of single-stranded RNA to recognize small molecules [6]. Simultaneously, Tuerk and Gold published an iterative procedure of an “in vitro” selection process called “SELEX” (Systematic Enhancement of Ligands by Exponential Enrichment) to select an specific RNA aptamer that target and inhibit the bacteriophage protein T4 DNA polymerase [7]. Aptamers are highly resistant to degradation and can be chemically synthesized at a relatively low cost with high reproducibility. They can be customized to incorporate chemical modifications and can be joined with affinity tags or labels. In 1999, Jayassena proposed the aptamers as rivals for antibodies [8]. The same year, our group described the target switching method to produce a synthetic oligonucleotide capable of recognizing protein phosphatase 2A (PP2A) in the histological section prepared from murine cerebellum [9]. This aptamer–antibody parity was subsequently demonstrated for other aptamers specific for the tumor suppressor protein Cpd1/ANP32e [10]. Soon later, fluorescent aptamers were used for the detection of Endothelial Regulatory Protein Pigpen [11], the mucin MUC-1 [12], and Anp32e [13,14]. DNA aptamers were shown to compete with antibodies for ERK2-protein in cells [15]. Finally, in 2010, the aptamers were formerly presented as a histochemical tool using CD30 aptamers as a probe [16].

On the other hand, DNAzymes peroxidase are complexes formed by oligonucleotides containing guanine quadruplex helix and hemin as prosthetic group, which catalyze the decomposition of hydrogen peroxide with the consequent substrate oxidation [17]. The hemin molecule can be conjugated to the 5′ end of DNA oligonucleotide in order to increase the peroxidase activity [18], and Sakharov analyzed the linker length effect on enzyme activity [19]. Dai showed the role of metal in catalytic activity [20] and the improvement of DNA analogues on the stability of the structured DNAzyme [21]. Compared to natural protein peroxidases, DNAzymes are small size molecules, easy to synthesize and manipulate. They can be rationally designed for allosteric control, presenting a powerful catalytic toolkit in biosensing, biomaterials, and bio-molecular devices.

In this article we used an aptamer with dual properties (to recognize the PTEN antigen + peroxidase activity), for two important reasons. First, PTEN (Phosphatase and Tensin Homolog Deleted on Chromosome 10) is the second most studied tumor suppressor after p53 because of its relevance in the clinical prognosis of cancer. The PTEN gene is most frequently mutated in human cancers, including melanoma, glioblastoma, prostate, breast, lung, ovary, and endometrial cancers [22,23,24,25]. Second, the absence of PTEN is a predictive marker for endometrial precancer [26], and thus serves as an excellent negative control for our aptamers. The PTEN protein has two domains, a phosphatase domain (PTPs) and the regulatory domain. Aptamers were developed using a synthetic peptide which recognizes the enzyme domain of phosphatase. In this model, aptamer PTENz7 competes with antibodies raised in rabbit serum using the same antigen [27].

In this work we describe the application of DNAzyme peroxidase joined to aptamers as a standardized tool to stain the PTEN tumor suppressor protein in murine brain, human colon, and endometrial hyperplasia and cancer specimens.

## 2. Materials and Methods

### 2.1. Reagents and Preparation

PTENz14-106, PTENz7-biotin, PTENz7-106, PTENz7-70, and PS2.M-H1 DNA aptamers were purchased from IDT (Coralville, IA, USA) and Macrogen (Seoul, Korea). Hemin and 3,3’-diaminobenzidine (DAB) were purchased from Sigma-Aldrich (St. Louis, MO, USA). IgG monoclonal PTEN antibodies (sc-20 PTEN Santa Cruz, CA, USA) and HRP-conjugated goat anti-rabbit IgG were purchased from Promega (Madison, WI, USA). Tris(hydroxymethyl) aminomethane (Tris buffer), potassium chloride, sodium chloride, ethylene-diamine-tetraacetic acid (EDTA), dimethyl sulfoxide (DMSO) and hydrogen peroxide 30% (H_2_O_2_), Tween 20%, absolute ethanol, and xylene were purchased from Biopack (Buenos Aires, Argentina), and Triton X-l00 from Anedra (Buenos Aires, Argentina). Synthetic balsam was purchased from Alwik (Buenos Aires, Argentina). All chemicals were reagent grade and used without further purification. All solutions were prepared with ultra-pure water (Milli-Q).

### 2.2. Preparation of DNA Oligomer Stock Solution

The lyophilized oligonucleotides were prepared as stock solution (100 µM) in TE buffer (10 mM Tris-HCl, 5 mM EDTA, pH 8) and stored at −20 °C. The DNA concentration was adjusted by absorbance measurement at 260 nm with a spectrophotometer (Nanodrop 2000, Thermo Fisher (Waltham, MA, USA)).

### 2.3. Tissue Specimens

Murine tissues were obtained from C57BL-6J inbreed at the animal facility of the National University of San Martin (UNSAM). The experiments were approved by the Local Ethical Committee for the Use of Experimental Animals, (CICUAE Aval No 04/2019). The tissues were sectioned at a 5-μm thickness and placed on charged slides.

Human samples, anonymized formalin-fixed, paraffin-embedded tissue blocks, were obtained from the archives of the Department of Pathology of the Hospital de Clínicas “José de San Martín”, School of Medicine, National University of Buenos Aires with institutional review board approval. These specimens were donated by patients with informed consents to the José de San Martín “Hospital de Clínicas” University Hospital, for research purposes.

### 2.4. Tissue Preparation

Histological preparations were made from endometrium and colon tissue samples obtained from healthy individuals and cancer-diagnosed patients. The tissue samples were fixed in 4% paraformaldehyde, embedded in paraffin, sliced, and mounted on silanized glass, preserved in paraffin. Paraffin removal was carried out by heating for 45 min at 62 °C two washes with xylene for 10 min each. Tissue slices were rehydrated by a sequential wash in 100%, 90%, and 70% ethanol for 5 min each. Antigen was retrieved by incubating the slices for 20 min at 90–95 °C in a citrate buffer pH = 6 containing 0.05% Tween-20. Endogenous peroxidase activity was blocked by using 3% hydrogen peroxide for 20 min. Blocking of nonspecific bindings was performed by incubation with 2% Bovine Serum Albumin (BSA) fraction V (Sigma-Aldrich, St. Louis, MO, USA) in phosphate buffer saline (PBS) or TKT buffers (25 mM pH 8 Tris-HCl, 20 mM KCl, 200 mM NaCl, 0.05% Triton X-100, 0.1% dimethyl-sulfoxide DMSO) for 30 min. Samples were incubated overnight at 25 °C (in a humidity chamber) with 100 µl of PTENz7-87 biotin aptamer or PTENz7-106 dual aptamer, PTENz7-70 dual aptamer, PS2.M-H1, each at a final concentration of 5 µM.

### 2.5. Immunohistochemistry

Tissue samples were incubated overnight at 4 °C with 100 µl of IgG monoclonal PTEN antibody, diluted 1/100 in PBS pH 7 (Monoclonal Anti-Human PTEN (clone 6H2.1), Cascade Bioscience, Cat # ABM-2052) and washed three times with PBS containing 0.2% of Tween 20 (PBS-T). A secondary antibody staining kit, including horseradish peroxidase (HRP)-conjugated horse anti-mouse IgG antibody, was purchased from Vector Laboratories (Burlingame, CA, USA). Rabbit antibody against PTEN peptide [27,28] was used to compete with aptamers for PTEN recognition. The tissue was incubated for one hour with a 1/200 dilution of goat anti-rabbit IgG-HRP conjugated antibody (Santa Cruz Biotechnology, Dallas, TX, USA).

### 2.6. Histochemistry

**Figure 1:**
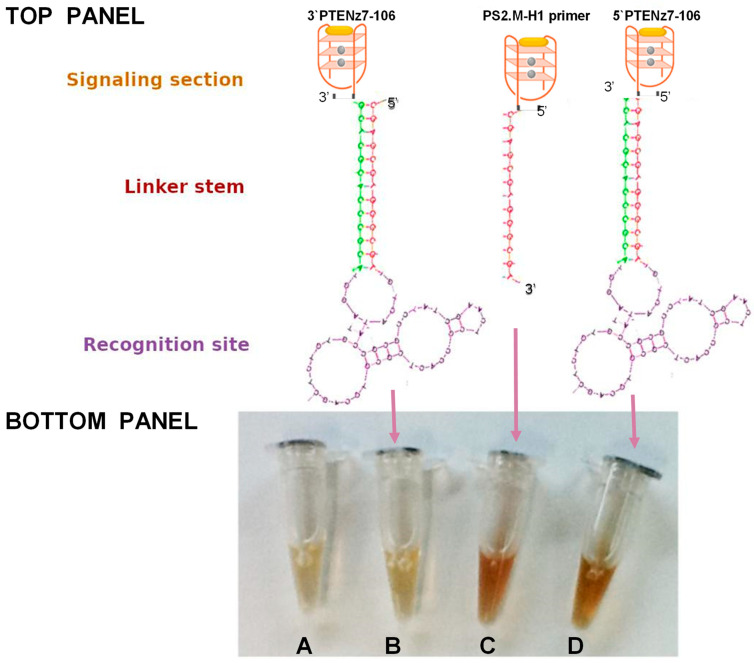
Top Panel: Schematic representation of a DNAzyme peroxidase-aptamer conjugates 3′PTENz7-106, PS2.M-H1, and 5′PTENz7-106. Gray circles represent potassium, and yellow oval hemin. Bottom Panel: Oligonucleotide peroxidase activity developed with 3,3’-diaminobenzidine (DAB) after 5 min in reaction containing the DNAzyme peroxidase-aptamer conjugates indicated by arrows (**B**–**D**). (**A**) = no DNAzyme (Control). The sequences of these DNAzyme/aptamers are shown in Table 1 using the same color code.

Intensity and patterns of the PTEN target was detected by using synthetic aptamers coupled to a biotin-avidin peroxidase system as previously described [14,27] or by using the PS2.M DNAzyme incorporated to aptamers as described in Figure 1. The aptamers were incubated in TKT buffer. Hemin was solubilized in dimethyl sulfoxide (DMSO) (1 mg/mL) and stored at -20 °C. Freshly prepared diluted solutions of hemin in TKT buffer were prepared prior to each experiment. The aptamer-PS2.M hemin complex, the biotin-aptamer, or the control PS2.M were diluted to a final concentration of 5 µM with TKT buffer containing hemin at final concentration of 100 µM. In order to obtain the adequate conformation of the aptamers, the mixture was heated at 95 °C for 5 min, quickly cooled at 4 °C for ten minutes, and incubated for 30 min at room temperature. After cooling, equal volumes of TKT buffer containing 2% of BSA were mixed and then used to cover the slices for 3 h at room temperature or overnight at 4 °C. The slices were then washed three times for 5 min each with TKT buffer.

### 2.7. Peroxidase Development

Peroxidase activity was detected by incubation with the chromogen 3,3′-diaminobenzidine tetrahydrochloride (DAB) solution (0.05% DAB, 0.015% H2O2, 0.01M PBS, pH 7.2) at room temperature until color development.

### 2.8. Mounting Tissue Sections

Tissues were washed three times with Milli-Q water and dehydrated by sequential washes in 70%, 90%, and 100% ethanol for 5 min each, then washed with xylene for 5 min. Nuclear counterstaining was carried out by using hematoxylin stain. Slides were mounted with synthetic balsam. Sample images were taken using a Leica DM2500 optical microscope and digitized with a Leica DFC295 camera.

## 3. Results

### 3.1. Addition of the PS2.M DNAzyme to the 5′ End of Single Strand DNA Maintains Its Peroxidase Activity

We first designed a dual aptamer, an aptamer with peroxidase activity and ability to recognize a specific target. This dual aptamer made only from single-stranded DNA consists of three parts (Figure 1, Top Panel): a recognition site (Aptamer) of the target. This region is single-stranded DNA formed by the 50 bases of the combinatorial sequences involved in the recognition site, a linker stem (primers). These constant regions contain the sequence of the primers, necessary for the amplification during the selection steps. The primers contain a region of 13 reverse complementary nucleotides, forming a fork with a constant and stable double helix with a melting temperature of 72 °C; and a signaling section (peroxidase DNAzyme) consisting of single strand DNA of 18 bases that forms a G-quadruplex with high affinity for hemin and thus, with peroxidase activity. This sequence named PS2.M was previously reported to have peroxidase activity [17].

In this study we designed two dual aptamers specific for PTEN (PTENz7-106 and PTENz14-106) (Figure 1). The recognition site specific for PTEN was obtained as previously described [27] from an aptamer library consisting of two regions in the DNA molecule of 87 mers. The sequences of these dual aptamer consisting of 106 bases (shown in Table 1) were custom ordered from (Macrogen, Seoul, Korea) and their peroxidase activity were tested and compared to another aptamer (5′primer PS2.M-H1) lacking the recognition site. All aptamers were used at 5 µM. As shown in Figure 1, controls (buffers + developing solutions without any aptamer) and the PTENz7-106 3′ dual aptamer did not show any peroxidase activity. In contrast the 5′ primer and the PTENz7-106 5′ dual aptamer demonstrated similar peroxidase activity.

These results indicated that addition of the stem site and the recognition site did not alter the activity of the 18 bases signaling site when attached to the 5′ end.

### 3.2. PTENz7-87 Aptamer Detects PTEN Protein in Human Endometrium Histological Sections

**Figure 2:**
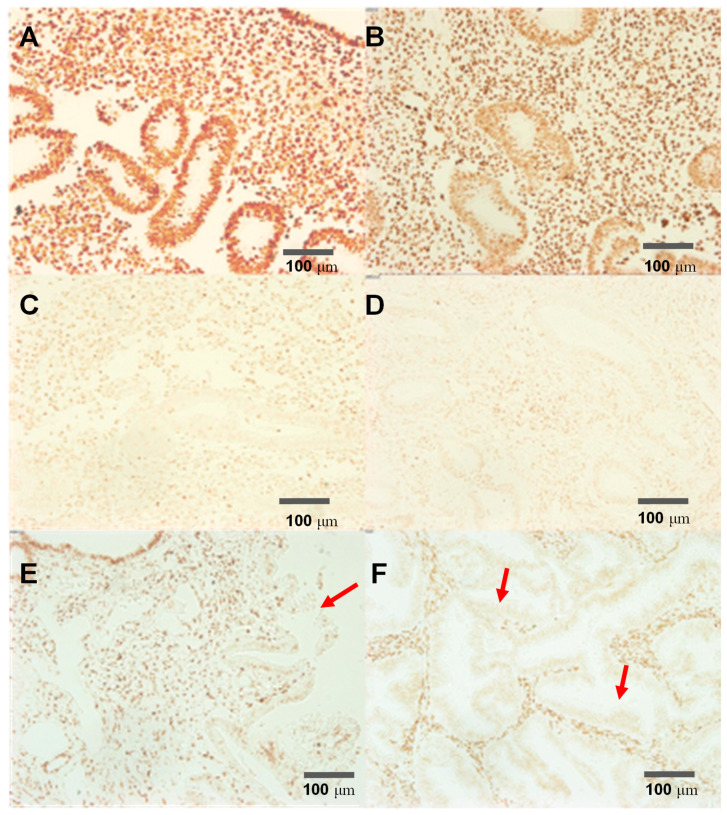
The PTENz7-87-biotin aptamer recognizes specifically the PTEN protein in human endometrial tissue. Endometrial samples were subjected to antigen retrieval and DNAse treatment and stained. (**A**) Control: Normal endometrial tissue stained using PTENz7-87-biotin and developed with avidin peroxidase; (**B**) Normal endometrial tissue stained using MAb 6H2 mouse monoclonal antibody; (**C**) Normal endometrial tissue stained using PTENz7-87-biotin competed with 5 μM of peptide; (**D**) Normal endometrial tissue stained using PTENz7-87-biotin competed with 2 mM GST-PTEN recombinant protein. (**E**) Hyperplastic tissue stained using PTENz7-87-biotin. The arrow shows an area with partial loss of PTEN stain in the adenoid. (**F**) Adenocarcinoma tissue stained using PTENz7-87-biotin. The arrows show examples of areas with loss of PTEN stain in all adenoids. Scale bars represent 100 μm.

In order to test the specificity of the recognition site of our construct we used an PTENz7-87 aptamer coupled to biotin in a panel of human endometrial tissue that included normal tissue (PTEN+), hyperplasic tissue previously confirmed as containing regions with loss of PTEN protein (PTEN-) and carcinoma tissue (PTEN-). Figure 2 shows that PTENz7-87 biotin aptamer detects PTEN protein in normal endometrium (Figure 2A) and hyperplasic tissue (Figure 2E) but not in adenocarcinoma tissue. Specificity was further confirmed by blocking with PTEN peptide (Figure 2C) and recombinant GST-PTEN protein (Figure 2D). Additionally, MAb 6H2 mouse monoclonal antibody produced a similar staining compared to PTENz7-87 biotin aptamer (Figure 2B).

### 3.3. The Dual DNAzyme/Aptamer Recognizes and Stain PTEN Protein in the Mouse Cerebellum in One Step

**Figure 3:**
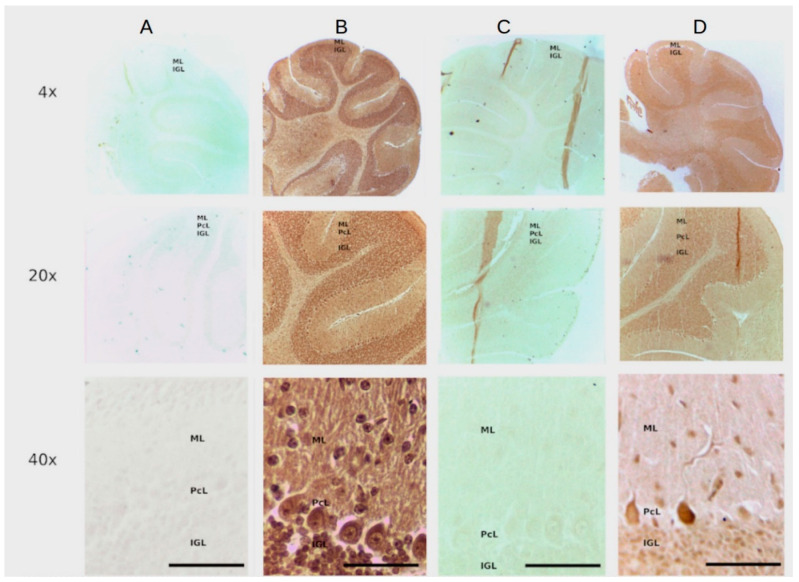
The PTENz7-106 and PTENz14-106 dual aptamers specifically detect PTEN protein in Mouse cerebellum. Column (**A**) Control sections stained with the PS2.M-H1. Column (**B**) Sections stained with the PTENz7-106 dual aptamer. Column (**C**) Sections preincubated with the PTEN rabbit antiserum and then stained with the PTENz7-106 dual aptamer. Column (**D**) Section preincubated with the PTEN rabbit antiserum and then stained with the PTENz14-106 dual aptamer. All samples were developed with DAB. The rows are at 10×, 20×, and 40× magnifications. ML: Molecular layer; PcL: Purkinje cells layer; IGL: Internal granular layer.

After confirming the specificity of the recognition site (PTEN z7-87 mers) in endometrial tissue using an avidin biotin system (see above Figure 2) we made a dual PTENz7-106 aptamer by adding this sequence to 3′ end of the PS2.M DNAzyme as shown in Figure 1. This dual PTENz7-106 aptamer was tested for its ability to recognize and simultaneously stain its specific PTEN target. We used histological sections prepared from mouse cerebellum for the following reasons: (i) The mouse cerebellum is an excellent model for PTEN studies because the subcellular localization of this protein can be easily observed in Purkinje cells, the largest neurons in the brain, as we previously demonstrated by the rabbit antiserum and aptamers that are used in this study [27]. (ii) The aptamer PTENz7-87 targets the first 7 amino acids present in a region of a 14 amino acids sequence conserved in both murine and human PTEN protein. (iii) The aptamer PTENz14-87 targets the last seven amino acids of this conserved sequence. The differences between the PTENz7 and the PTENz14 were previously described in Moncalero et al. [27] (See Figure 3 therein). These experiments were carried out with PS2.M DNAzyme or dual aptamer in TKT buffer that contains hemin (100 μM). It is important to mention that the DMSO (at 0.1%) is critical to allow the solubilization of hemin. As shown in Figure 3 (columns A and B), the dual 5′DNAzyme/aptamer-PTENz7-106 produced a strong signal compared to controls (incubated with the 5′ primer PS2.M-H1, 39 mers). This result demonstrates that only a dual DNAzyme/aptamer containing the recognition site was able to detect PTEN. In addition, when the sections were preincubated with a rabbit antiserum that recognizes the same recognition site of the PTEN protein detected by the dual 5′DNAzyme/aptamer-PTENz7-106 the signal was lost (column C). The rabbit antiserum that recognizes a target region different did not block the signal from the one detected dual 5′ aptamer PTENz14-106 [27]. The aptamers against PTEN show both distributions, in the molecular layers as well as in the internal granular layer, a lower intensity of stain can be observed for dual aptamer PTENz14-106. These results are in agreement with previous data obtained using aptamers PTEN biotin-avidin peroxidase system [27].

To summarize, this novel dual aptamer was able to simultaneously detect and stain with high specificity the PTEN protein in mouse cerebellum histological sections.

### 3.4. The Dual DNAzyme/Aptamer without the Stem Linker Simultaneously Recognize and Stain PTEN Protein in Colon and Endometrial Cancer

**Figure 4:**
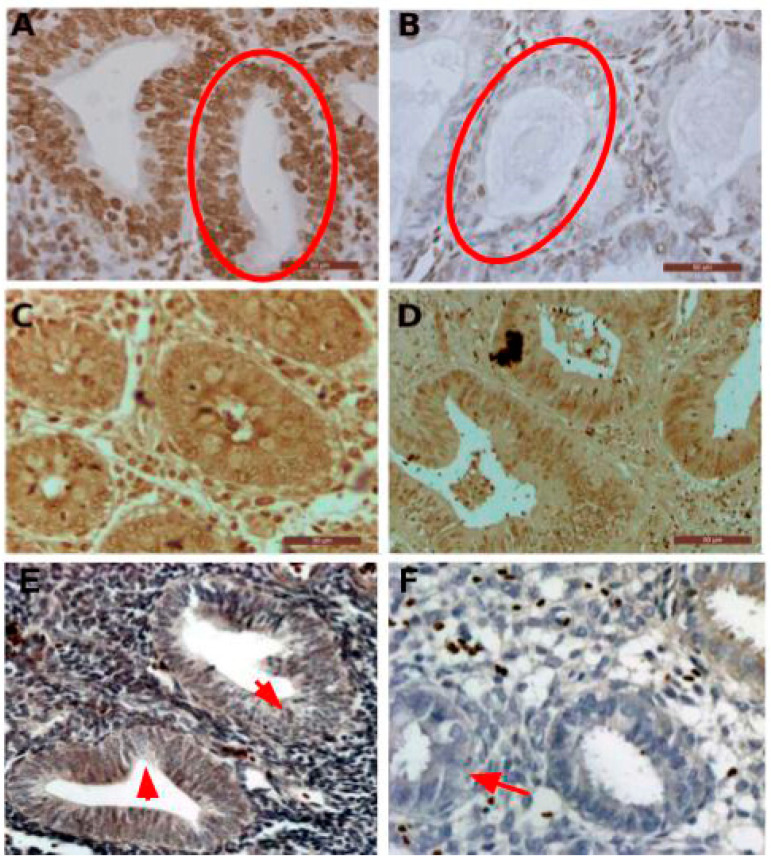
The PTENz7-70 dual aptamer specifically detects PTEN protein in normal and neoplasic endometrial and colon samples. (**A**) Human non-neoplasic endometrial tissue treated for antigen retrieval and stained using the reduced PTENz7-70 dual aptamer. Nuclei were counterstained with hematoxylin stain. The circle indicates PTEN^+^ acinar cells. (**B**) Human hyperplasic tissue stained using the reduced PTENz7-70 dual aptamer counterstained with hematoxylin. The circle indicates PTEN^−^ acinar cells (**C**) Normal human colon tissue treated with PTENz7-70 dual aptamer. Counterstain was omitted. (**D**) Human colon adenocarcinoma stained with PTENz7-70 dual aptamer. (**E**,**F**) The arrows in the figures show examples of areas in hyperplasic tissue with partial loss of PTEN (classified as PTEN**^+/−^**) signal in acini. Scale bars represent 50 μm.

To test the hypothesis that only the recognition site and the signaling section are sufficient for the dual activity we designed a shorter PTENz7 dual aptamer (PTENz7-70 aptamer, full sequence shown in Table 1) lacking 36 mers the STEM linker. The PTENz7-70 dual aptamer was tested in colon and endometrium histological sections. This shorter dual DNAzyme/aptamer showed similar detection and staining capabilities compared to the original 106 mers dual aptamer. Figure 4A shows that PTENz7-70 aptamer produced a robust signal in acinar cells from non-neoplasic human endometrial tissue. However, as expected, the PTENz7-70 dual aptamer did not detect PTEN protein in acinar cells from hyperplasic endometrial tissue samples previously confirmed as PTEN- by antibodies (Figure 4B). The PTENz7-70 dual aptamer produced a robust staining in non-neoplasic as well neoplasic human colon samples (Figure 4C,D) previously confirmed as PTEN+ by antibodies.

To validate these results in a larger number of samples the PTENz7-70 dual aptamer was tested in 60 specimens from endometrial tissue and compared with the signal produced with the monoclonal antibody MAb 6 H2. As summarized in Table 2, both PTENz7-70 dual aptamer and the monoclonal antibody MAb 6 H2 showed identical results. Figure 4E,F shows examples of hyperplasic endometrial tissues with partial loss of PTEN classified as PTEN+/−.

## 4. Discussion

Since the pioneering work of Ellington in 1990 aptamers against different small molecules have been developed. The detection of these small molecules such as theophylline [29], melanin and dopamine [30] was achieved by creating an RNA aptamer-based electrochemical biosensor for solution-based assays. However, the range of detection in solution based assays is limited by the background produced by the hemin. To overcome this limitation, Zhang et al., successfully used SYBR Green I [31]. The diagnosis of specific cancer types is based on information obtained from histological sections of biopsy tissues [32,33] that reveals the tissue morphology and the presence of specific tumor markers in cells and subcellular compartments [34]. Despite the growing use of aptazymes as biosensors, as far as we know, dual aptamers have not been developed for histology. Although it has been reported that the activity of an aptamer that recognizes Mucin 1 when linked to the 18 mers PS2.M (MUC1-A15-PS2.M aptamer) increases its peroxidase activity in solution [21] the use of this construct has not been reported for histological staining. In that article the authors noted that the affinity of PS2.M for hemin (the prosthetic group) has a dissociation constant of 27 ± 3 nM [21] and the hemin concentration must be above ~30 nM to maintain an active enzymatic complex. To ensure that in solution all aptamer molecules form a complex with hemin it is necessary that the hemin:aptamer ratio in solution should be at least 100:1. For an aptamer at 5 μM, the hemin concentration should be 100 μM. We achieved this high concentration of hemin by adding DMSO in the TKT buffer (see Materials and methods, 2.6 section). When we added 1% DMSO in the incubation solutions we observed in our staining a robust signal with a clear background.

In this work, we have designed a dual aptamer (see Figure 1 and accompanying text) capable of recognizing its PTEN protein target and produce a robust and reproducible staining signal from murine and human histological sections. The staining pattern obtained with this dual aptamer was in agreement with results previously obtained with these aptamers using a biotin/avidin-peroxidase system in the murine brain [27] as well as with PTEN rabbit antiserum [28]. The murine tissue was used to take advantage of a known system previously described in our lab in which we utilized a PTEN rabbit antiserum against the same peptide used to develop the aptamer [27,28]. Our present data confirms that the dual aptamer preserves its ability to recognize the selected region of the PTEN protein as well as its catalytic activity.

The specificity of the recognition site for the PTEN protein has been confirmed by (a) competition of the PTEN recognition site with the targeted peptide and the recombinant full PTEN protein (Figure 2), (b) masking the PTEN protein by blocking with a rabbit PTEN antiserum made against the same peptide used as immunogen (Figure 3), and (c) by using known PTEN- samples (hyperplasic tissue) as negative controls (Figure 2). Cross-reactivity of the dual aptamer with genomic DNA was ruled out because PTEN staining was not affected by DNAse I treatment of the samples. We next showed that a shorter dual PTEN aptamer (without the stem linker; PTENz7-70) gave identical results when used in histological sections prepared from human endometrial and colon tissues (Figure 4A–F). The results were reproduced and validated by comparing with the MAb 6H2 in a panel of 60 human specimens (Table 2).

**Figure 5:**
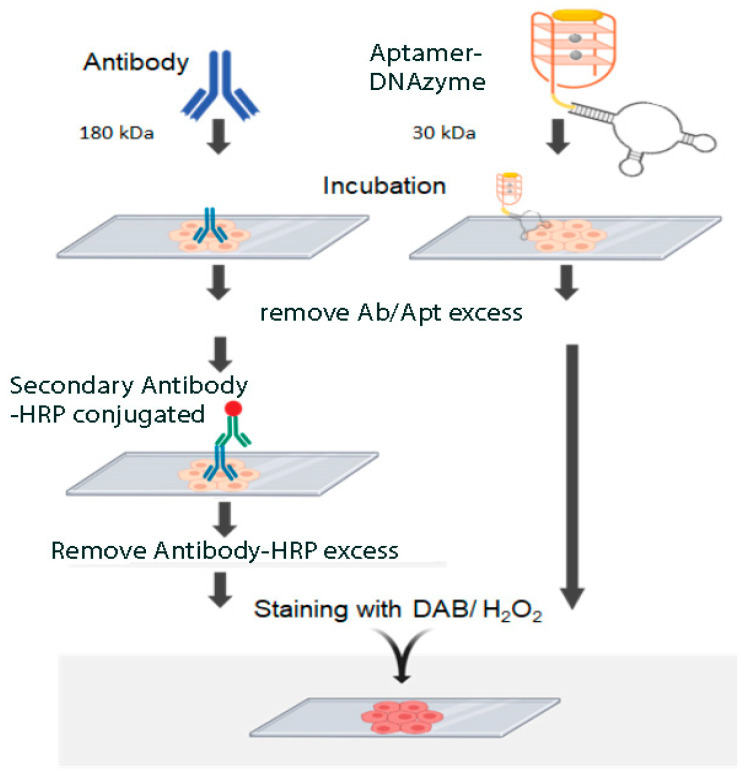
Comparison of immunohistochemistry with antibodies and histochemistry with Aptamer-DNAzyme conjugates.

Our dual aptamer allowed the detection of the PTEN protein in all typical or atypical hyperplasia as well as in adenocarcinoma of human endometrium. This system may allow a more precise quantitation since the aptamer:enzyme ratio is 1:1 and therefore the signal is directly proportional to the amount of target protein. Another advantage of this detection system is the DNA nature that allows its storage and transfer at room temperature. Nucleic acids can be synthetically obtained with a high level of standardization, opening a range of possibilities for the development of fast, simple, and reliable detection methodologies. Figure 5 summarizes the advantages of dual aptamers compared to traditional antibody staining: The use of dual aptamers requires less steps and reagents making the entire process faster and cheaper. In addition, global access to the sequences of these aptamers allows unlimited and reproducible sources of detection system that is not possible with traditional antibodies due to for instance batch to batch differences and limited production [8].

In this study we showed that the shorter PTENz7-70 aptamer displayed identical robust and specific signal compared to its larger PTENz7-106 version (Figure 4). While this is an obvious advantage in terms of production cost and synthesis efficiency it may not be always possible because a shorter version of the PTENz14-106 (PTENz14-70) failed to produce a staining signal.

## 5. Conclusions

In summary we reported for the first time the design, construction, and application of a synthetic dual aptamer able to specifically detect the PTEN protein in histological murine and human paraffin sections. Moreover, this system produced identical results when compared to the antibody MAb 6H2 that is the gold standard to detect PTEN for diagnosis of endometrial pathology.

## Figures and Tables

**Table 1:** DNA oligonucleotides used in the construction of the aptamer-DNAzyme, aptamer-HRP and DNAzyme peroxidase. Color code: Orange = PS2.M; Red and Green = 5′ and 3′ STEM linker; **Black/Purple** = target recognition site.

Aptamer	Sequence
Dual 5′ Aptazyme PTENz14 106	**5′-GTGGGTAGGGCGGGTTGGCCGGAATTCCGAGCGTGGGCGTGAGCCCTAAACACAAGTCCGCAGGGGTGTGGTAATATTCGCAGTTGTGTGTACGCCCACGCTCGAG-3′**
PTENz7 87-biotin	**Biotin 5′-CGGAATTCCGAGCGTGGGCGTGGTCATACCGCGCCTATCGAACTCGCCACTCGCGTGCAGCTCTGTGTAGGTACGCCCACGCTCGAG-3′**
Dual 5′Aptazyme PTENz7 106	**5′-GTGGGTAGGGCGGGTTGGCCGGAATTCCGAGCGTGGGCGTGGTCATACCGCGCCTATCGAACTCGCCACTCGCGTGCAGCTCTGTGTAGGTACGCCCACGCTCGAG-3′**
Dual 3′Aptazyme PTENz7 106	**5′-CGGAATTCCGAGCGTGGGCGTGGTCATACCGCGCCTATCGAACTCGCCACTCGCGTGCAGCTCTGTGTAGGTACGCCCACGCTCGAGCGTGGGTAGGGCGGGTTGG-3′**
dual Aptazyme-PTENz7 70	**5′-GTGGGTAGGGCGGGTTGGCGGTCATACCGCGCCTATCGAACTCGCCACTCGCGTGCAGCTCTGTGTAGGT-3′**
PTENz7 51-biotin	**Biotin 5′-GGTCATACCGCGCCTATCGAACTCGCCACTCGCGTGCAGCTCTGTGTAGGT-3′**
PS2.M-H1	**5′-GTGGGTAGGGCGGGTTGGCCGGAATTCCGAGCGTGGGCGT-3**

**Table 2:** The PTENz7-70 dual aptamer reproduced the signal obtained with Mab-6H2 monoclonal specifically in a panel of neoplasic and hyperplasic endometrium tissue. The presence of PTEN in all acini was indicated as positive (+). The absence of PTEN in all ducts was stated as minus (−). Partial loss of PTEN were indicated as (+/−) in hyperplasic tissue. The results show that both the PTENz7-70 dual aptamer and theMAb-6H2 produce identical a signal with identical intensity and pattern in a panel of 60 samples.

*PTEN*	Typical Hyperplasia *n* = 18			Atypical Hyperplasia *n* = 24			Adenocarcinoma *n* = 18		
**stain**	**+**	**+/−**	**−**	**+**	**+/−**	**−**	**+**	**+/−**	**−**
**PTENz7-70**	10	6	2	7	11	6	-	3	15
**MAb-6 H2**	10	6	2	7	11	6	-	3	15

## Data Availability

The data presented in this study are available on request from the corresponding author. Some human data are not public available due to data protection reasons.
